# Histogram analysis of continuous-time random walk and restrictive spectrum imaging for identifying hepatocellular carcinoma and intrahepatic cholangiocarcinoma

**DOI:** 10.3389/fonc.2025.1516995

**Published:** 2025-03-10

**Authors:** Bo Dai, Yihang Zhou, Lei Shen, Hanhan Li, Ting Fang, Jiayin Pan, Yan Wang, Wei Mao, Xiaopeng Song, Fengshan Yan, Meiyun Wang

**Affiliations:** ^1^ Department of Radiology, Henan Provincial People’s Hospital & Zhengzhou University People’s Hospital, Zhengzhou, China; ^2^ Department of Radiology, Xinxiang Medical University People's Hospital & Henan Provincial People's Hospital, Zhengzhou, China; ^3^ Department of Radiology, West China School of Public Health and West China Fourth Hospital, Chengdu, China; ^4^ Central Research Institute, United Imaging Healthcare, Shanghai, China; ^5^ Biomedical Research Institute, Henan Academy of Sciences, Zhengzhou, China; ^6^ Laboratory of Brain Science and Brain-Like Intelligence Technology, Institute for Integrated Medical Science and Engineering, Henan Academy of Sciences, Zhengzhou, China

**Keywords:** continuous-time random walk, restrictive spectrum imaging, whole-lesion histogram, hepatocellular carcinoma, intrahepatic cholangiocarcinoma

## Abstract

**Background:**

To compare the ability and potential additional value of various diffusion models, including continuous-time random walk (CTRW), restrictive spectrum imaging (RSI), and diffusion-weighted imaging (DWI), as well as their associated histograms, in distinguishing the pathological subtypes of liver cancer.

**Methods:**

40 patients with liver cancer were included in this study. Histogram metrics were derived from CTRW (D, α, β), RSI (f_1_, f_2_, f_3_), and DWI (ADC) parameters across the entire tumor volume. Statistical analyses included the Chi-square test, independent samples t-test, Mann-Whitney U test, ROC, logistic regression, and Spearman correlation.

**Results:**

Patients with hepatocellular carcinoma exhibited higher values in f_1 median_, f_1 20th_, f_1 40th_, and f_1 60th_ compared to patients with intrahepatic cholangiocarcinoma, whereas D_mean_, D_median_, D_40th_, D_60th_, and D_80th_ percentiles were lower (P<0.05). Among the individual histogram parameters, f_1 40th_ percentile demonstrated the highest accuracy (AUC = 0.717). Regarding the combined and single models, the total combined model exhibited the best diagnostic performance (AUC = 0.792). Although RSI showed higher diagnostic efficacy than CTRW (AUC = 0.731, 0.717), the combination of CTRW and RSI further improved diagnostic performance (AUC = 0.787), achieving superior sensitivity and specificity (sensitivity = 0.72, specificity = 0.80).

**Conclusion:**

CTRW, RSI, and their corresponding histogram parameters demonstrated the ability to distinguish between pathological subtypes of liver cancer. Moreover, whole-lesion histogram parameters provided more comprehensive statistical insights compared to mean values alone.

## Introduction

Liver cancer is one of the leading causes of cancer-related deaths globally, with incidence and mortality rates steadily increasing (1, 2). Hepatocellular carcinoma (HCC) is the most common form of primary liver cancer, accounting for 90% of cases, while intrahepatic cholangiocarcinoma (ICC) makes up 10-15% (1). Compared to HCC, ICC is more aggressive and has a higher potential for metastasis, resulting in differences in treatment and prognosis (3, 4). On dynamic contrast-enhanced computed tomography (CT) or magnetic resonance imaging (MRI), ICC can present imaging features similar to those typical of HCC, making differentiation complex and time-consuming, even for experienced specialists (5, 6). Additionally, the use of contrast agents is costly and may be contraindicated in certain patients (7). A biopsy is also a common method for distinguishing pathological subtypes of liver cancer. However, this approach is invasive and has several drawbacks, including low patient compliance and a high rate of complication. Thus, the development of a non-invasive technique to accurately differentiate pathological subtypes of liver cancer remains a significant challenge.

DWI, the pioneering diffusion imaging technique employed in clinical practice, quantifies the extent of restricted diffusion motion of water molecules through the quantitative parameter known as the apparent diffusion coefficient (ADC) (8). Study has demonstrated that ADC can serve as a potential surrogate imaging biomarker for distinguishing HCC, ICC, and metastatic cancer (9). However, DWI assumes a Gaussian distribution of the diffusion motion of water molecules in biological tissues, which makes it unable to fully capture the non-Gaussian characteristics of water diffusion in complex subcellular microstructures, which in turn leads to limitations in the accuracy of ADC value assessment (10). To obtain more accurate information on water diffusion and to map tissue microstructure, researchers have developed non-Gaussian mathematical models based on high b-value DWI, such as the continuous-time random walk (CTRW) (11, 12). The CTRW provides three parameters: diffusion coefficient (D), temporal diffusion heterogeneity (α), and spatial diffusion heterogeneity (β). The D describes the non-Gaussian diffusion behavior in biological tissues, while α and β are related to temporal and spatial diffusion heterogeneity, respectively. Both parameters may reflect different aspects of tissue structural heterogeneity within the voxel (13). Currently, CTRW has demonstrated significant potential in distinguishing benign from malignant breast lesions, identifying pathological subtypes, and evaluating prognosis (14–16). However, reports on the use of CTRW to assess the severity of liver fibrosis and liver cancer metastasis are limited (12, 17). Various DWI techniques encounter the challenge of significant overlap between diffusion signals due to the mixed signals from intracellular and extracellular water molecule diffusion (18). A novel diffusion-weighted magnetic resonance imaging technique, known as restriction spectrum imaging (RSI), addresses this issue (19). In the three-compartment model, each compartment represents a different water molecule diffusion, f_1_ indicates the signal fraction of restricted diffusion, reflecting the tumor’s cellular composition; f_2_ represents the signal fraction of hindered diffusion, indicating delayed water molecule passage around cellular obstacles; and f_3_ represents the signal fraction of free water diffusion, reflecting microcirculation perfusion (20). RSI can isolate areas of truly restricted diffusion by separating and removing hindered diffusion signals, offering a more direct measurement of tumor cells compared to other diffusion-weighted methods (19). RSI has achieved significant breakthroughs in distinguishing benign from malignant tumors and has demonstrated potential in differentiating cancerous from non-cancerous tissues in studies involving lungs, prostate, and breast (18, 20, 21). However, the relative value of CTRW and RSI in distinguishing pathological subtypes of liver cancer has not been compared. Histogram is a classical analysis method based on image voxel values, which not only has high reproducibility and consistency, but also can provide additional quantitative indicators (22–24). Therefore, if histogram analysis is applied to RSI and CTRW, it is expected to mine richer image information and thus provide more evidence for ICC and HCC identification.

The objective of this study was to compare the value of RSI, CTRW and DWI and their associated histograms in differentiating HCC from ICC, with the aim of finding an accurate, non-invasive imaging marker to guide clinical decision making.

## Materials and methods

### Participants

**Figure 1:**
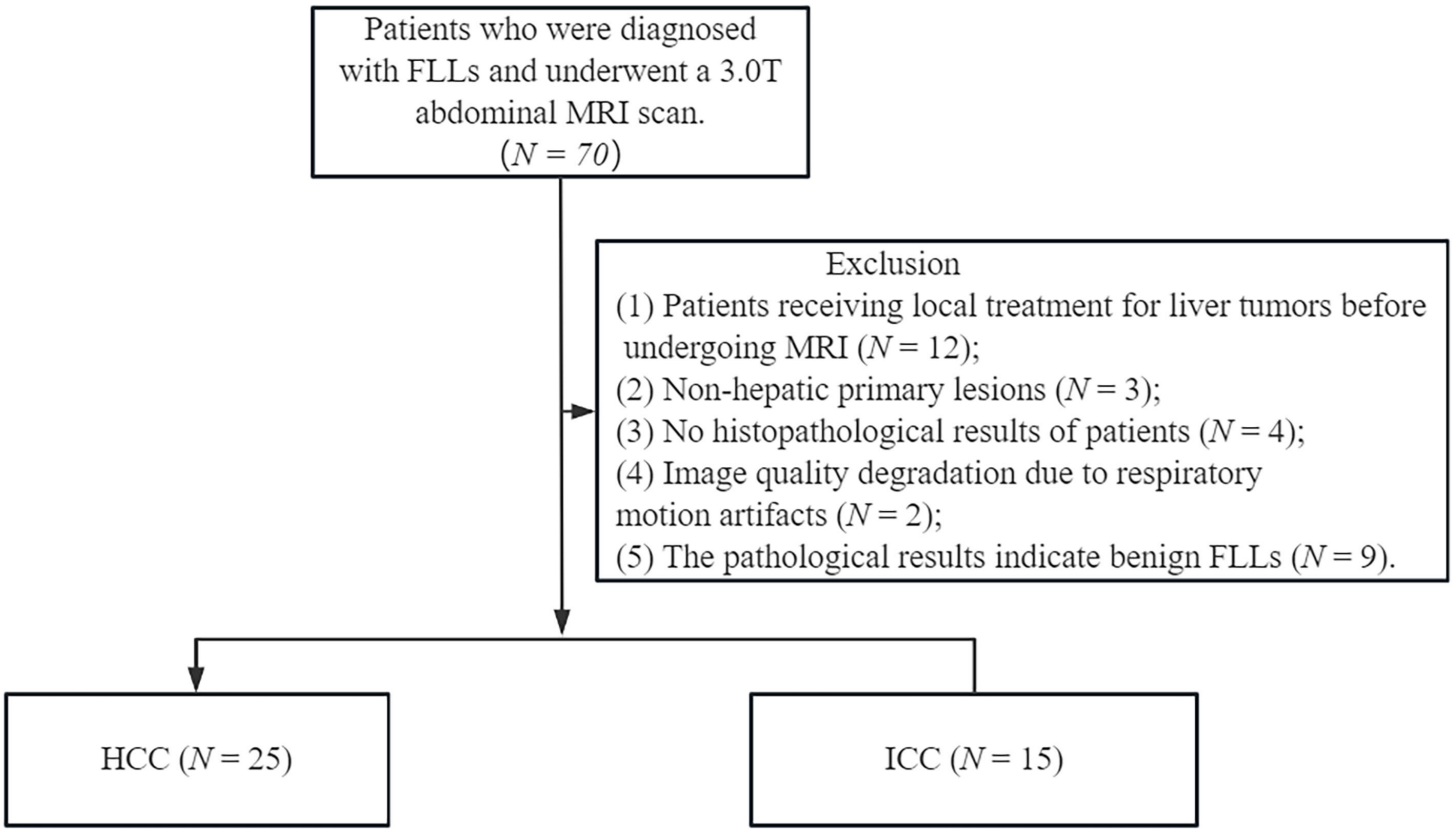
Flow diagram of the study population.

This prospective study received approval from the Research Ethics Committee and obtained written informed consent from all participating patients. From March 2022 to March 2024, a total of 70 patients diagnosed with focal liver lesions (FLLs) underwent liver MRI examinations. Patient demographic data were collected from electronic medical records. Inclusion criteria are as follows: (1) patients with pathologically confirmed HCC or ICC; (2) those without MRI contraindications, such as cardiac pacemakers, ferromagnetic implants, or claustrophobia. exclusion criteria are as follows: (1) patients who had undergone prior local treatment for liver tumors, such as resection, transplantation, chemotherapy, trans arterial chemoembolization, radiofrequency ablation, or immunosuppressive therapy; (2) patients with non-liver primary lesions and those whose image quality was compromised by ghosting, distortion artifacts, or respiratory motion artifacts, making it impossible to delineate the FLLs. The flowchart for this selection process is illustrated in **Figure 1**.

### Image acquisition

All initially enrolled patients underwent MRI examinations using a 3.0 T MRI scanner (uMR790, United Imaging Healthcare, Shanghai, China) equipped with a 12-channel phased-array body coil. Patients were positioned in a supine, head-first orientation. Initially, routine axial T_1_-weighted and axial and coronal T_2_-weighted images were acquired. Subsequently, diffusion-weighted images with multiple b-values were obtained. The detailed MRI parameters are provided in **Table 1**.

**Table 1:** MR Scanning Parameters.

Parameter	T_1_WI	T_2_WI	T_2_WI	Multiple b-value DWI
TR (ms)	3.98	4190	4190	3051
TE (ms)	1.48	93.60	97.20	92.30
Flip angle (deg.)	12	90	90	90
Slice thickness (mm)	5.0	6.0	6.0	5.0
FOV*(mm^2^)	400×280	380×280	380×380	320×200
Matrix	320×320	243×304	228×304	160×160
Bandwidth (kHz)	1260	700	700	1670
NEX	1	1	1	1,1,1,1,1,1,1,1,4,4,6,8,10,12
b values (s/mm^2^)	/	/	/	0,25,50,100,150,200,400,600,800,1000,1500,2000,3000
Orientation	Axial	Axial	Coronal	Axial
Breath control	Breath holding	Breath holding	Breath holding	Breathe freely
Scanning time	15.8s	33.5s	33.5s	2.49min

T_2_WI, T_2_-weighted imaging; T_1_WI, T_1_-weighted imaging; DWI, diffusion weighted imaging; FOV, field of view; NEX, number of excitations; TE, echo time; TR, repetition time.

### Data post-processing

The parameter from different DWI techniques were calculated using prototype software developed with Python (Python 3.8; Python Software Foundation). These calculations were based on the following formulas.

(1) The mono-exponential model:


$$S_{b}/S_{o}=\exp(-b\;x\;ADC)$$

In this model, *S*_0_ refers to the signal obtained using a b-value of 0 mm²/s, while S_b_ corresponds to the signal obtained using a b-value of 800 mm²/s. ADC denotes the apparent diffusion coefficient (25).

(2) The CTRW model:


$$S_{b}/S_{o}=E_{\alpha }[-{\left(b\;x\;D\right)}^{\beta }]$$

In this model, *E* . dotes the Mittag-Leffler function of order α. D represents an anomalous diffusion coefficient, while α and β are diffusion metrics associated with temporal and spatial diffusion heterogeneity, respectively. Both α and β range from 0 to 1, indicating the degree of homogeneity within the medium (14).

(3) The Tri-Compartmental RSI model:


$$\frac{S_{b}}{S_{o}}=f1\;x\;\exp(-b\cdot D_{1})+f_{2}\;x\;\exp(-b\cdot D_{2})+f_{3}\;x\;\exp(-b\cdot D_{3})$$

In this model, the signal intensities *S*_b_ and *S*_0_ correspond to specific b–values, with b = 0 mm²/s D_1_, D_2_, and D_3_ represent the diffusion coefficients for restricted, hindered, and free diffusion, respectively. The variables f_1_, f_2_, and f_3_ denote the volume fractions of these diffusion components. Restricted diffusion refers to water molecules trapped within intracellular spaces, resulting in very slow diffusion. Hindered diffusion occurs when extracellular water molecules are obstructed by cells, impeding their movement Free diffusion, by contrast, describes the rapid, unrestricted movement of water molecules. Based on reference studies, the optimal diffusion coefficients for D_1_, D_2_, and D_3_ were determined to be 0.05 × 10^−3^ mm^2^/s, 1.25 × 10^−3^ mm^2^/s, and 20 × 10^−3^ mm^2^/s, respectively. These values were used to calculate f_1_, f_2_, and f_3_ maps. The b-values used for fitting the RSI model were 0, 25, 50, 100, 150, 200, 400, 600, 800, 1000, 1500, 2000, and 3000 s/mm^2^ (26).

**Figure 2:**
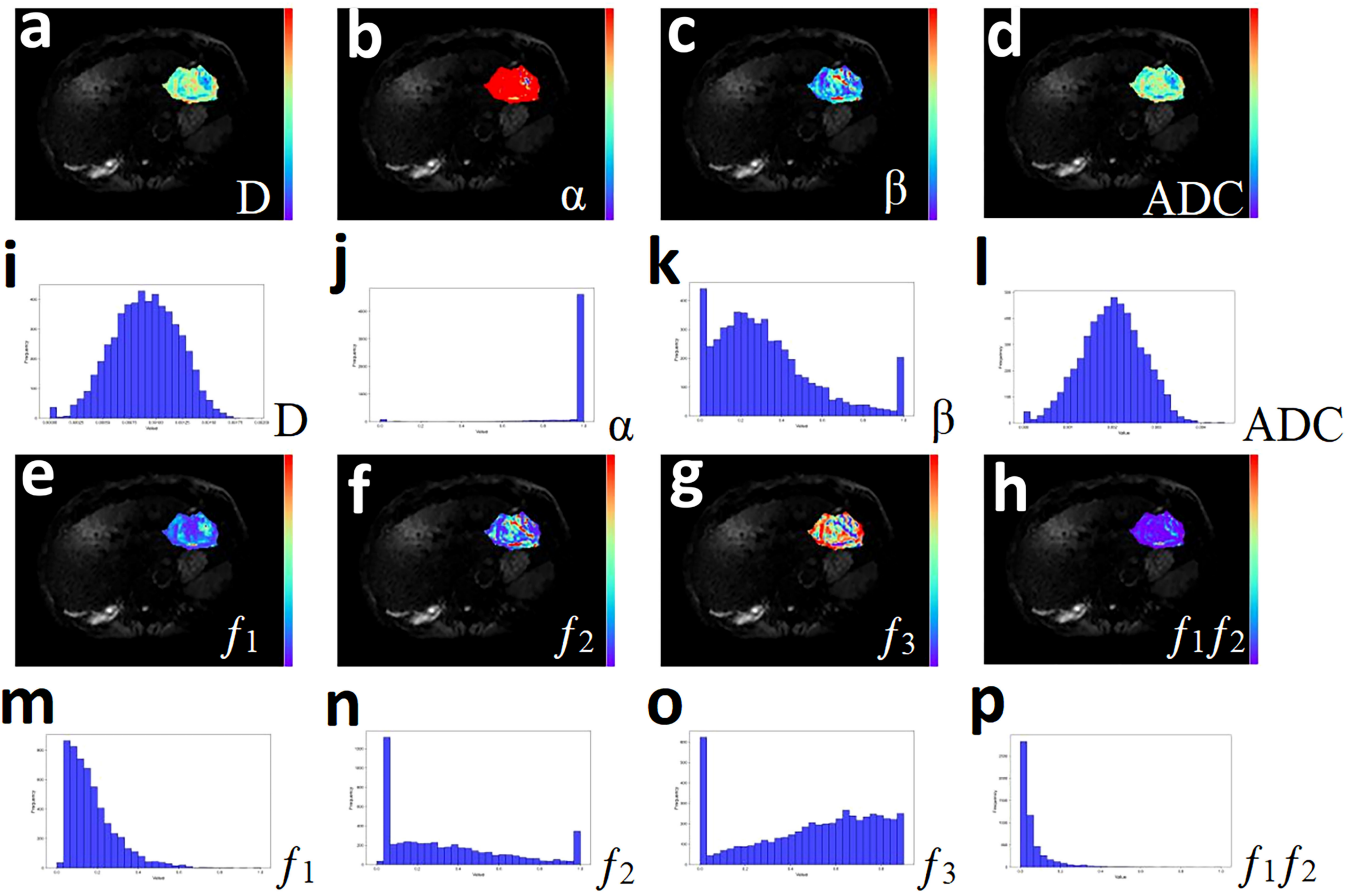
A 51-year-old male with hepatocellular carcinoma. **(a-h)** Pseudo-color drawings corresponding to D, α, β, and ADC. **(i-p)** Histograms of D, α, β, and ADC.

**Figure 3:**
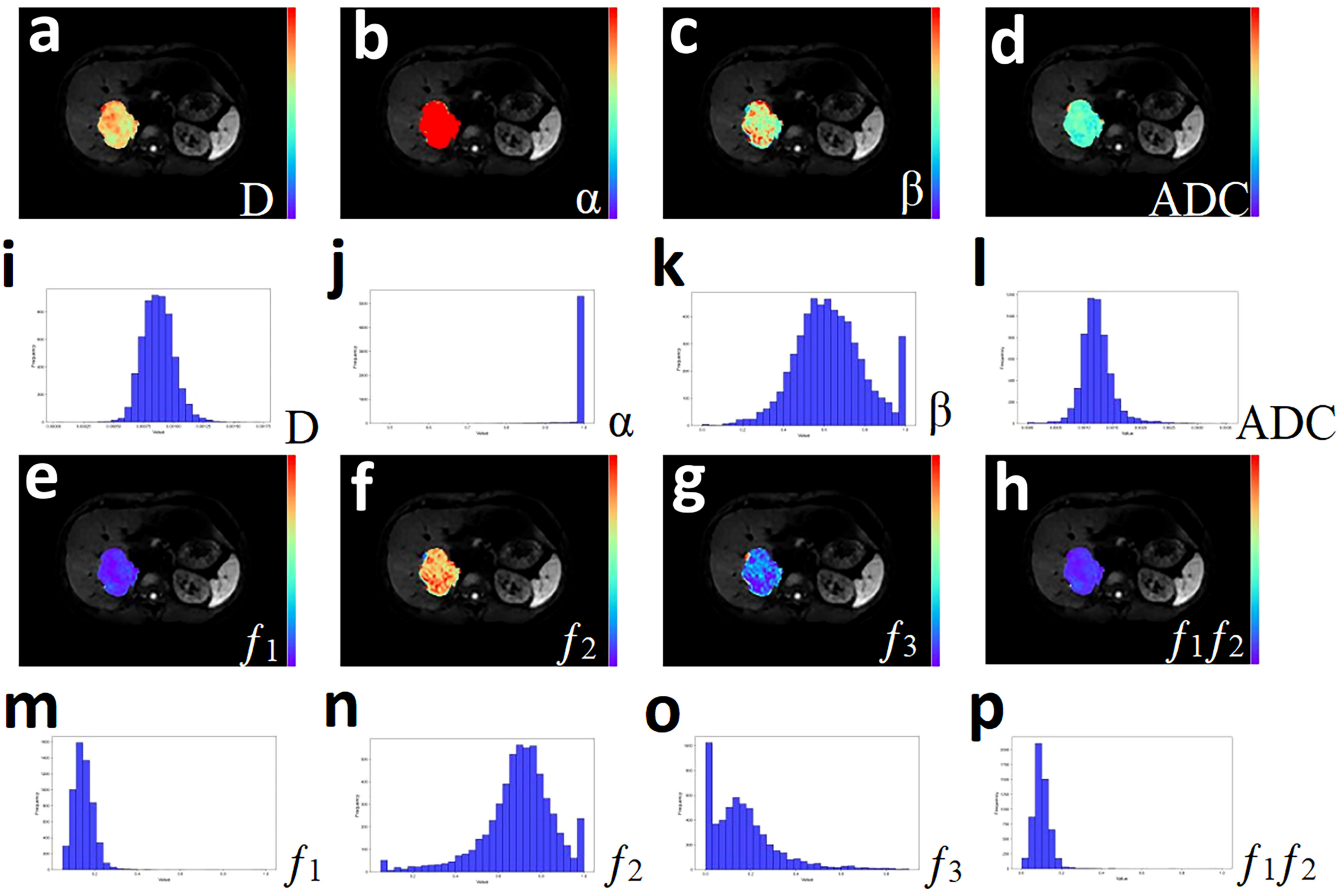
A 33-year-old male with Cholangiocarcinoma. **(a-h)** Pseudo-color drawings corresponding to D, α, β, and ADC. **(i-p)** Histograms of D, α, β, and ADC.

In order to fully reflect the lesion information, all slices containing tumors were selected from DWI images with b = 600 mm²/s, using conventional T_1_-weighted and T_2_-weighted images as references, and regions of interest (ROIs) were manually outlined layer by layer along the edges of the tumors. This process should avoid obvious bleeding, necrosis and other areas as much as possible, and the final volumes of interest (VOIs) were composed of different slices of ROI. Subsequently, VOIs were replicated on D, α, β, f_1_, f_2,_ f_3_, and ADC pseudo color maps, and the following histogram indicators were also extracted based on existing studies: mean, median, maximum, minimum, 20th percentile, 40th percentile, 60th percentile, 80th percentile, standard deviation, variance, kurtosis, and skewness (23, 27). **Figures 2** and **3** present representative MRI images of HCC and ICC, respectively. The above work was performed collaboratively by two radiologists (with 8 and 14 years of experience in abdominal imaging diagnosis) who had no prior knowledge of the clinical and pathological data, and in the event of disagreement, the decision was taken by negotiation and ultimately by the more experienced radiologist.

### Statistical analysis

All data were statistically analyzed using MedCalc 15.0 (MedCalc Software, Mariakerke, Belgium) and SPSS 26.0 (SPSS, Chicago, IL). The Shapiro-Wilk test and Levene’s test were employed to assess the normality of general data and histogram parameters, as well as the equality of variances. The Chi-square test, independent samples t-test, and the Mann-Whitney U test were used to compare differences in general data and quantitative histogram parameters between the HCC and ICC groups. Logistic regression was applied to establish combined models of histogram parameters and various model parameters. Receiver operating characteristic (ROC) curves were used to evaluate the ability of individuals and combined to distinguish pathological subtypes of liver cancer. Diagnostic thresholds, sensitivity, and specificity were determined based on the maximum Youden index. Spearman correlation analysis was conducted to explore correlations between parameters, with correlation coefficients (r) categorized as follows: 0-0.49 for poor correlation, 0.50-0.74 for moderate correlation, and 0.75-1.00 for strong correlation (11). A significance level of *P<* 0.05 was considered statistically significant for all analyses.

## Results

### Patient characteristics

Initially, 70 patients diagnosed with FLLs were enrolled in this prospective study. After excluding 30 patients due to preoperative treatment (*N* = 12), non-hepatic primary lesions *(N* = 3), unclear histopathological results (*N* = 4), poor image quality (*N* = 2), and pathological results indicating benign FLLs (*N* = 9), a total of 40 patients were included in the final analysis. The 40 patients had an average age of 57.43 ± 10.47 years (range: 33-75 years). Based on histopathological results, 25 patients were diagnosed with HCC and 15 patients were diagnosed with ICC. **Table 2** presents the detailed patient characteristics.

**Table 2:** Demographics.

	HCC	ICC	*P*
Number of patients	25	15	/
Gender (M:F)	20:5	11:4	0.705
Age (year) mean ± SD	58.04 ± 9.96	56.4 ± 11.55	0.638
AFP (ng/ml) Median (IQR)	59.85 (6.55, 3740.00)	4.13 (2.73, 14.35)	0.011*
Background liver			
Chronic hepatitis B	21	6	0.006*
Cirrhosis	22	6	0.003*

SD, standard deviation; AFP, Alpha-Fetoprotein; *, *P* < 0.05; HCC, hepatocellular carcinoma; ICC, intrahepatic cholangiocarcinoma.

### Correlation between the mean values of CTRW-, RSI- and ADC-Derived Parameters

Spearman correlation analysis revealed several correlations, notably, D_mean_ and f_1 mean_ exhibited a strong negative correlation (r = -0.869, *P<* 0.0001), while D_mean_ and ADC _mean_ showed a strong positive correlation (r = 0.887, *P<* 0.0001). A moderate negative correlation was observed between f_1 mean_ and ADC _mean_ (r = -0.716, *P<* 0.0001), and similarly between α_mean_ and f_1 mean_ (r = -0.698, *P<* 0.0001). Furthermore, moderate positive correlations were found between D_mean_ and f_3 mean_ (r = 0.556, *P<* 0.0001), β_mean_ and f_2 mean_ (r = 0.661, *P<* 0.0001), as well as between f_3 mean_ and ADC _mean_ (r = 0.749, *P<* 0.0001). Other correlations among the parameters were not statistically significant.

### Comparison of histogram-derived parameters between HCC and ICC

**Figure 4:**
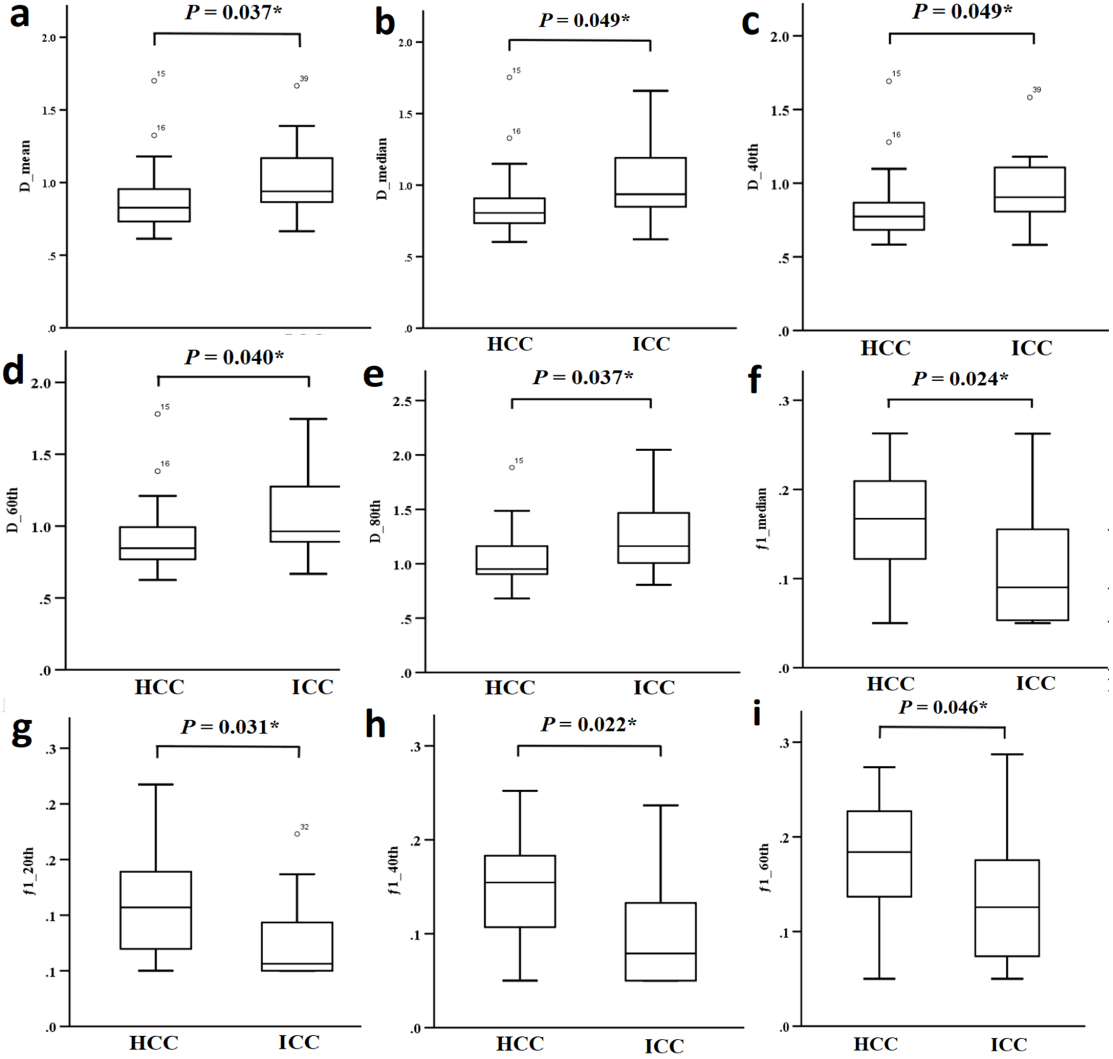
**(a-i)** Comparison of different parameters (D _mean_, D _median_, D _40th_, D _60th_, D _80th_, f_1 median_, f_1 20th_, f_1 40th_ and f_1 60th_) between the HCC group and the ICC group.

D_mean_, D_median_, D_40th_, D_60th_ and D_80th_ were significantly lower in HCC patients compared to ICC patients (P = 0.037, 0.049, 0.049, 0.040, and 0.037, respectively). Additionally, f_1 median_, f_1 20th_, f_1 40th_, and f_1 60th_ were significantly higher in HCC patients compared to ICC patients (P = 0.024, 0.031, 0.022, and 0.046, respectively). No significant differences were observed in the other parameters, as shown in **Table 3** and **Figure 4**.

**Table 3:** Comparison of Histogram-Derived Parameters between HCC and ICC.

Parameter	HCC ( N = 25 ) Median (IQR)	ICC ( N = 15 ) Median (IQR)	Z	P
D _mean_	0.827 (0.730, 0.979)	0.939 (0.860, 1.196)	2.081	0.037
D _median_	0.805 (0.729, 0.958)	0.936 (0.831, 1.216)	1.970	0.049
D _40th_	0.773 (0.675, 0.916)	0.904 (0.803, 1.107)	1.970	0.049
D _60th_	0.846 (0.765, 1.021)	0.963 (0.878, 1.330)	2.053	0.040
D _80th_	0.951 (0.893, 1.168)	1.162 (0.977, 1.583)	2.081	0.037
*f*_1 median_	0.167 (0.103, 0.215)	0.090 (0.050, 0.162)	2.253	0.024
*f*_1 20th_	0.106 (0.599, 0.147)	0.056 (0.050, 0.109)	2.152	0.031
*f*_1 40th_	0.154 (0.090, 0.188)	0.079 (0.050, 0.134)	2.290	0.022
*f*_1 60th_	0.184 (0.121, 0.242)	0.125 (0.063, 0.180)	1.999	0.046

HCC, hepatocellular carcinoma; ICC, intrahepatic cholangiocarcinoma; IQR, Interquartile Range; D, diffusion coefficient; *f*_1,_ the signal fraction of restricted diffusion; D _mean_, D _median_, D _40th_, D _60th_ and D _80th_ represent the mean, median, 40th percentile, 60th percentile and 80th percentile of the D in the continuous-time random walk, respectively; *f*_1 median_, *f*_1 20th_, *f*_1 40th_ and *f*_1 60th_ represent the median, 20th percentile, 40th percentile and 60th percentile of the *f*_1_ in the Tri-Compartmental restriction spectrum imaging model, respectively. D _mean_, D _median_, D _40th_, D _60th_ and D _80th_ are expressed in units of ×10^-3^ square millimeters per second (mm^2^/s); *f*_1 median_, *f*_1 20th_, *f*_1 40th_ and *f*_1 60th_ are unitless. Mann-Whitney U test was used for group comparison.

### Diagnostic performance of histogram-derived parameters in distinguishing pathological subtypes of liver cancer

**Figure 5:**
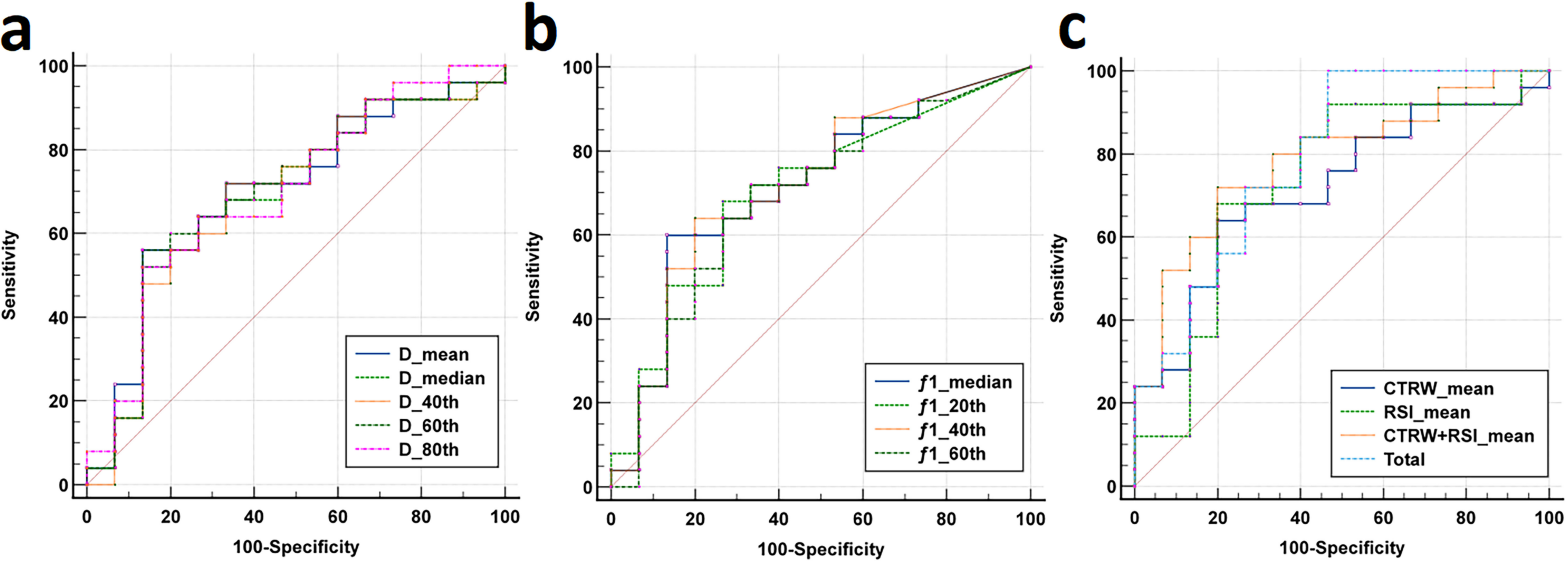
**(a-b)** The ROC curves for CTRW-derived histogram parameters (D _mean_, D _median_, D _40th_, D _60th_, D _80th_) and RSI-derived histogram parameters (f_1 median_, f_1 20th_, f_1 40th_, f_1 60th_) distinguishing between the HCC group and the ICC group. **(c)** The ROC curves for CTRW, RSI, the combined model (CTRW+RSI) and the total combined model (D _mean_, D _median_, D _40th_, D _60th_, D _80th_, f_1 median_, f_1 20th_, f_1 40th_ and f_1 60th_) distinguishing between the HCC group and the ICC group.

Individual histogram parameters D_mean_, D_median_, D_40th_, D_60th_, D_80th_, f_1 median_, f_1 20th_, f_1 40th_, and f_1 60th_, along with combined models - total (a combination of D_mean_, D_median_, D_40th_, D_60th_, D_80th_, f_1 median_, f_1 20th_, f_1 40th_, and f_1 60th_), CTRW combined model (D, α, and β), RSI combined model (combining f_1_, f_2_, and f_3_), and CTRW+RSI combined model - all demonstrated statistically significant ROC curves. AUC values of total, f_1 40_^th^, f_1 median_, f_1 20_^th^, D_80th_, D_mean_, D_60th_, f_1 60th_, D_40th_, and D_median_ were 0.792, 0.717, 0.715, 0.703, 0.699, 0.699, 0.696, 0.691, 0.688, and 0.688, respectively. The combined models demonstrated significantly higher diagnostic performance, and among individual parameters, the combined models showed the best sensitivity. Regarding the CTRW, RSI, and CTRW+RSI combined models, their diagnostic efficiencies in distinguishing HCC and ICC were ranked as follows: AUC (CTRW+RSI) > AUC (RSI) > AUC (CTRW), with AUC values of 0.787, 0.731, and 0.717, respectively. Although RSI exhibited better diagnostic performance than CTRW in differentiating the pathological subtypes of liver cancer, the combination of CTRW and RSI improved diagnostic efficiency, providing the highest sensitivity and specificity (**Table 4**, **Figure 5**).

**Table 4:** ROC Analysis of the Diagnostic Performance for Different Parameters and Methods Alone or in Combination for Distinguishing HCC from ICC.

Parameters	AUC(95% CI)	Cut off	Sensitivity(95% CI)	Specificity(95% CI)	Youden Index	*P*
D_mean_ (×10^-3^)	0.699 (0.533-0.833)	0.831	0.560 (0.349-0.756)	0.867 (0.595-0.983)	0.427	0.037*
D_median_ (×10^-3^)	0.688 (0.522-0.825)	0.805	0.520 (0.313-0.722)	0.867 (0.595-0.983)	0.387	0.049*
D _40th_ (×10^-3^)	0.688 (0.522-0.825)	0.826	0.720 (0.506-0.879)	0.667 (0.384-0.882)	0.387	0.049*
D _60th_ (×10^-3^)	0.696 (0.530-0.831)	0.857	0.560 (0.349-0.756)	0.867 (0.595-0.983)	0.426	0.040*
D _80th_ (×10^-3^)	0.699 (0.533-0.833)	0.950	0.520 (0.313-0.722)	0.867(0.595-0.983)	0.386	0.037*
*f*_1 median_	0.715 (0.550-0.846)	0.162	0.600 (0.387-0.789)	0.867 (0.595-0.983)	0.467	0.025*
*f*_1 20th_	0.703 (0.537-0.836)	0.077	0.680 (0.465-0.851)	0.733 (0.449-0.922)	0.413	0.034*
*f*_1 40th_	0.717 (0.553-0.848)	0.133	0.640 (0.425-0.820)	0.800 (0.519-0.957)	0.440	0.023*
*f*_1 60th_	0.691 (0.525-0.827)	0.154	0.720 (0.506-0.879)	0.667 (0.384-0.882)	0.387	0.046*
Total	0.792 (0.634-0.904)	0.579	1.000 (0.863-1.000)	0.533 (0.266-0.787)	0.533	0.002*
CTRW	0.717 (0.553-0.848)	0.353	0.640 (0.428-0.820)	0.800(0.519-0.957)	0.440	0.023*
RSI	0.731 (0.567-0.858)	0.379	0.680 (0.465-0.851)	0.800 (0.519-0.957)	0.480	0.016*
ADC_mean_(×10^-3^)	0.637 (0.470-0.783)	1.199	0.600 (0.387-0.789)	0.733 (0.449-0.922)	0.333	0.150
CTRW+RSI	0.787 (0.628-0.900)	0.318	0.720 (0.506-0.879)	0.800 (0.519-0.957)	0.520	0.003*

ROC, Receiver Operating Characteristic; HCC, hepatocellular carcinoma; ICC, intrahepatic cholangiocarcinoma; AUC, Area Under the Curve; CI, Confidence Interval; D, diffusion coefficient; *f*_1,_ the signal fraction of restricted diffusion; D _mean_, D _median_, D _40th_, D _60th_ and D _80th_ represent the mean, median, 40th percentile, 60th percentile and 80th percentile of the D in the continuous-time random walk, respectively; *f*_1 median_, *f*_1 20th_, *f*_1 40th_ and *f*_1 60th_ represent the median, 20th percentile, 40th percentile and 60th percentile of the *f*_1_ in the Tri-Compartmental restriction spectrum imaging model, respectively; *, *P* < 0.05; CTRW, continuous-time random walk; RSI, restriction spectrum imaging; ADC, apparent diffusion coefficient. D _mean_, D _median_, D _40th_, D _60th_ and D _80th_ are expressed in units of ×10^-3^square millimeters per second (mm^2^/s); *f*_1 median_, *f*_1 20th_, *f*_1 40th_ and *f*_1 60th_ are unitless.

## Discussion

This study represents a significant and pioneering effort in evaluating the diagnostic capabilities of two advanced, non-Gaussian diffusion-weighted models, CTRW and RSI, in distinguishing HCC from ICC. The results demonstrated that both the CTRW and RSI models effectively distinguish between HCC and ICC. Notably, the RSI model exhibited superior diagnostic performance compared to the CTRW, with the signal fraction of restricted diffusion (f_1_) from RSI showing greater potential for differentiating HCC from ICC. Moreover, combining histogram parameters or integrating multiple diffusion models significantly enhanced diagnostic accuracy. The combined histogram parameter model demonstrated the highest diagnostic efficacy among all approaches tested. This study establishes the potential of these models to improve diagnostic accuracy and provides a foundation for future research into their broader clinical application, particularly in the context of liver cancer subtyping.

A substantial body of research suggests that the ADC values derived from the mono-exponential model are based on the assumption that water molecule diffusion follows a Gaussian distribution (28). However, the actual microenvironment within biological tissues, especially in heterogeneous tumors, is far more complex. In such environments, the movement of water molecules is restricted by cellular structures and membranes, resulting in non-Gaussian diffusion behavior (11). Consequently, ADC values fail to account for the non-Gaussian characteristics of water diffusion within these intricate subcellular microstructures, limiting their diagnostic efficacy (12). This aligns with our study’s findings, which indicate that ADC values are suboptimal for distinguishing between pathological subtypes of liver cancer.

The RSI separates the diffusion of water molecules in tissues into restricted diffusion, hindered diffusion, and free water diffusion, and its parameters f_1_, f_2_, and f_3_ represent the proportions in which the above three compartments are located, typically summing to 1. In this study, the f_1 median_, f_1 20th_, f_1 40th_, and f_1 60th_ values were significantly higher in HCC patients compared to ICC patients, which may be related to the fact that HCC usually have higher cell densities and more tightly packed cells (29). Also in this study, we found that none of the differences in f_2_ and f_3_ between HCC and ICC were statistically significant. This is similar to the study by Xiong et al. (30), and we hypothesize that this may be related to the fact that the fitting of f_2_ and f_3_ values is susceptible to the number and size of b-values.

The CTRW model has three quantitative parameters, namely, D, α, and β. The rate parameter D is mainly used to reflect the speed of the diffusion of water molecules. The present work found that the D values (including D_mean_, D_median_, D_40th_, D_60th_, and D_80th_) were significantly lower in HCC patients. One possible explanation is that HCC is characterized by a higher cell density and more complex microstructure, which restricts the free movement of water molecules. In contrast, the central region of ICC consists of loose fibrous tissue, with tumor cells predominantly located at the periphery, often arranged into adenoidal patterns. This structural arrangement in ICC facilitates the diffusion of water molecules (31). The α and β describe the potential of water molecules to be retained or released during diffusion. There were no significant differences in both α and β between HCC and ICC in this study, which is not consistent with previous study (16). We consider that this may be related to the small sample size in this study and the different fitted b values used between studies.

This investigation also explore the correlations between mean histogram-derived parameters from these advanced models. The Spearman correlation analysis revealed a strong negative correlation between D_mean_ and f_1 mean_, a strong positive correlation between D_mean_ and ADC _mean_, and a moderate negative correlation between f_1 mean_ and ADC _mean_, indicating their association with tissue cellular structures (32). These findings support the consistency of these parameters in assessing tumor tissue characteristics. Theoretically, smaller α and β values suggest a more heterogeneous spatial environment (33), while f_1_, f_2_, and f_3_ correspond to intracellular restricted water, extracellular restricted water, and freely diffusing water, respectively (20). Further analysis demonstrated a moderate negative correlation between α _mean_ and f_1 mean_, as well as a moderate positive correlation between β_mean_ and f_2 mean_. This suggests that as cellular density increases, water molecules encounter more obstacles or irregularities during their movement between cells, leading to a greater proportion of water molecules being confined to intracellular spaces. Consequently, this results in an increase in the fraction of restricted diffusion (f_1_) and a decrease in the fraction of extracellular restricted water (f_2_). The observed correlations between diffusion parameters not only support the theoretical associations between specific parameters in these different diffusion models, but also have the potential to provide markers for tumor characterization assessment, such as monitoring cell morphology and density changes during treatment by f1-f2-ADC alterations. However, there are few relevant studies yet, and further large sample size experimental observation is still needed in the future.

In this study, the results indicate that both CTRW and RSI outperform traditional DWI-ADC in differentiating between HCC and ICC, with RSI exhibiting the highest discriminatory power. This superiority is likely due to the non-Gaussian distribution characteristics of CTRW and RSI, which, unlike the Gaussian assumptions of the ADC model, accurately reflect the complex diffusion behavior of water molecules and the intricate microstructure of tumor tissues (13). In addition, the high diagnostic performance of RSI may be attributed to the ability to isolate genuinely restricted diffusion areas, thereby minimizing interference from extracellular diffusion signals (34). This allows RSI to more directly measure tumor cell density and tissue structure, making the difference in high cell density regions between HCC and ICC more pronounced. Although CTRW and RSI represent significant advancements in DW-MRI, no single imaging technique currently fulfills all diagnostic requirements for tumors. Our study demonstrates that both the combined models of CTRW and RSI, as well as the integrated models of histogram parameters showing statistically significant differences between the pathological subtypes of liver cancer, provide superior diagnostic performance compared to individual models or histogram parameters used in isolation. This enhanced performance is attributed to the comprehensive integration of multidimensional information, including cellular metabolism and water molecule diffusion, which provides valuable complementary insights. Therefore, where possible, adopting multimodal imaging approaches for lesion assessment is likely to yield the greatest diagnostic benefits. By leveraging the strengths of various imaging techniques, clinicians can obtain a more holistic understanding of tumor characteristics, ultimately leading to more accurate diagnoses and better patient management.

## Limitations

Firstly, it was a single-center and relatively small sample size study Secondly, no test-retest procedure was implemented for diffusion MRI within the same participants. The Quantitative Imaging Biomarkers Alliance advocates for such retest procedures to assess the repeatability and reproducibility of quantitative MRI techniques. Thirdly, the scans in this study were performed a single imaging vendor or scanner. These limitations suggest that while our findings are promising, caution should be exercised in generalizing the results until further research can provide more robust validation. In the future, we will include more patients and conduct multicenter studies at different institutions, and will further optimize the parameters, port the relevant scanning protocols to different devices, and conduct external validation of the relevant parameter measurements with a view to improving the fitness and reducing the bias.

## Conclusion

In summary, both the CTRW and RSI models, along with their derived histogram parameters, demonstrated the ability to differentiate between pathological subtypes of liver cancer, whether utilized individually or in combination. Furthermore, whole-lesion histogram parameters provide richer statistical information compared to mean values, offering a quantitative approach to analyzing subtle changes in tumor voxels. This advancement has the potential to find an accurate, noninvasive imaging marker for the differentiation of HCC and ICC, which could help reduce unnecessary biopsies, especially in resource-limited settings, and in turn guide clinical decision-making.

## Data Availability

The raw data supporting the conclusions of this article will be made available by the authors, without undue reservation.
